# Investigations as to the Cause of the Great Gravity of Anthrax and Furuncles of the Face

**Published:** 1871-05

**Authors:** 


Investigations as to the Cause of the Great Gravity of Anthrax and Furuncles of the Face.—The numbers of the *Archives Generales de Medecine* for June, July, and August last, contain an elaborate article on this subject, by J. L. Reveratin, Interne Lauréat des Hôpitaux. The following are his conclusions:—1. Anthrax and furuncle of the face are very grave affections. 2. This gravity is owing to their being prone to be complicated with phlebitis. 3. Facial phlebitis tends to a fatal termination, either by its extending to the sinus of the dura mater, or by becoming the source of purulent infection. 4. Anthrax in the lips is complicated more frequently than when situated in other parts of the face with phlebitis, which fact is to be accounted for by the particular structure of the lips. 5. Anthrax of the lips is entirely different from malignant pustule. 6. The extension of phlebitis to the orbit, shown by the presence of exopthalmia, shows almost positively that the sinus has become affected. 7. Incision, made as rapidly and as largely as possible, appears to be the best means of preventing and sometimes of arresting the complication of phlebitis.

